# Suppurative Inguinal Lymphadenitis Secondary to Group A Streptococcal Vaginitis: A Case Report

**DOI:** 10.7759/cureus.92981

**Published:** 2025-09-22

**Authors:** Takahiro Nishiyama, Yusuke Yamaga, Keiichi Nagai, Shoichiro Okazaki

**Affiliations:** 1 Hematology, Ichinomiya Municipal Hospital, Ichinomiya, JPN; 2 Diagnostic Radiology, Ichinomiya Municipal Hospital, Ichinomiya, JPN

**Keywords:** abscess drainage, bacterial vaginitis, group a streptococcus, inguinal lymphadenopathy, suppurative lymphadenitis

## Abstract

Group A *Streptococcus* commonly colonizes the upper respiratory tract and skin but rarely causes bacterial vaginitis in non-pregnant women. Such an infection may progress to invasive disease via lymphatic spread. We report the case of a woman in her 50s, without significant medical history, who presented with green vaginal discharge and received empirical treatment with intravaginal metronidazole suppositories. Therapy was discontinued after four days due to menstruation, and a subsequent vaginal culture grew *Streptococcus pyogenes*. Immediately after menstruation, she developed a rapidly enlarging, painful left inguinal mass. Computed tomography revealed marked lymph node enlargement with central low attenuation, consistent with an abscess. Ultrasound-guided aspiration yielded purulent fluid positive for *Streptococcus pyogenes*. She was treated with intravenous ceftriaxone, followed by oral amoxicillin-clavulanate and then amoxicillin, for a total duration of four weeks, resulting in complete resolution without recurrence. This case highlights that group A *Streptococcus* can be a potential cause of acute inguinal lymphadenitis in non-pregnant women, particularly after genital symptoms, and emphasizes the importance of early diagnosis via aspiration and culture as well as prompt targeted antibiotic therapy and drainage to prevent invasive progression.

## Introduction

Group A *Streptococcus* (GAS) is a gram-positive pathogen that causes various human diseases, from mild superficial infections, such as pharyngitis and impetigo, to life-threatening invasive conditions, including necrotizing fasciitis and streptococcal toxic shock syndrome [1]. GAS most commonly colonizes the upper respiratory tract and skin. It can also infect the female genital tract, particularly during the peripartum period, potentially leading to severe invasive disease [2]. However, GAS-associated bacterial vaginitis in non-pregnant women is a rare and often underrecognized condition [3].

Recent evidence has shown that GAS can spread from colonized superficial sites, such as the skin and mucous membranes, to deeper tissues via lymphatic dissemination [4]. Nonetheless, previous reports have not documented purulent inguinal lymphadenitis caused by GAS after a genital tract infection.

Herein, we report a rare case of *Streptococcus pyogenes* (GAS) vaginitis complicated by purulent inguinal lymphadenitis in a healthy, non-pregnant woman. This case shows that it is important to consider GAS in the differential diagnosis of acute inguinal lymphadenitis, particularly when preceded by genital symptoms. It also emphasizes the need for early microbiological diagnosis via abscess aspiration and drainage, followed by prompt antimicrobial therapy, to prevent invasive progression.

## Case presentation

A woman in her 50s, with no significant previous medical or family history, no known drug allergies, and no pet exposure, initially presented to a local gynecology clinic due to a chief complaint of green vaginal discharge. The patient was diagnosed with bacterial vaginitis, and intravaginal metronidazole suppositories were prescribed, without awaiting vaginal secretion culture results. The treatment was discontinued after four days due to menstruation onset. Subsequent culture of the vaginal secretions grew *S. pyogenes*.

Immediately after the discontinuation of metronidazole and the end of the menstrual period, the patient noticed a painful, thumb-sized mass in her left inguinal region. At a local internal medicine clinic, she was diagnosed with lymphadenitis and prescribed with antipyretic analgesics. Over the next six days, the mass enlarged rapidly to the size of a clenched fist, with worsening pain that persisted despite treatment with acetaminophen at a dose of 3,000 mg/day (1,000 mg three times daily) and loxoprofen at a dose of 180 mg/day (60 mg three times daily). The pain disrupted the patient's sleep and caused gait disturbance, prompting her to visit our institution.

Upon hospital arrival, the patient's vital signs were as follows: temperature 37.3°C, blood pressure 120/76 mmHg, and pulse rate 100 beats/min. Physical examination revealed a tender, 8-cm mass in the left inguinal region, with overlying erythema and warmth but without evidence of skin thinning. Pain was exacerbated by walking, and intermittent limping was observed. As shown in Table 1, the laboratory tests revealed a normal white blood cell count (7,900/μL) with neutrophil predominance and lymphopenia (870/μL), an elevated C-reactive protein (CRP) level (6.25 mg/dL), and normal renal and hepatic function.

**Table 1:** Laboratory findings at the diagnosis of suppurative inguinal lymphadenitis WBC: white blood cells; RBC: red blood cells; MCV: mean corpuscular volume; MCH: mean corpuscular hemoglobin; MCHC: mean corpuscular hemoglobin concentration; PT: prothrombin time; APTT: activated partial thromboplastin time; FDP: fibrin/fibrinogen degradation products; TSH: thyroid-stimulating hormone; FT4: free thyroxine; ALP: alkaline phosphatase; ALT: alanine transaminase; AST: aspartate aminotransferase; CPK: creatine phosphorus kinase; LDH: lactate dehydrogenase; γ-GTP: gamma-glutamyl transpeptidase; T-CHO: total cholesterol; CHE: cholinesterase; BUN: blood urea nitrogen; UA: uric acid; TP: total protein; T-Bil: total bilirubin; ESR: erythrocyte sedimentation rate; CRP: C-reactive protein; C3: complement component 3; C4: complement component 4; sIL2R: soluble interleukin-2 receptor; RF: rheumatoid factor; ASO: antistreptolysin O; ACE: angiotensin-converting enzyme; HIV: human immunodeficiency virus; CMV: cytomegalovirus; EBV VCA: Epstein-Barr virus viral capsid antigen; EBNA: EBV nuclear antigen; T-SPOT.TB: t-spot tuberculosis test

Test	Result	Reference range
WBC (/μL)	7900	3300-8600
Neutrophil (%)	78	38-80
Lymphocyte (%)	11	16-49
Eosinophil (%)	1	0-8
Basophil (%)	1	0-2
Monocyte (%)	7	2-10
RBC (10⁶/μL)	3.93	3.86-4.92
Hemoglobin (g/dL)	12.3	11.6-14.8
Hematocrit (%)	36.7	35.1-44.4
MCV (fL)	93.4	83.6-98.2
MCH (pg)	31.3	27.5-33.2
MCHC (g/dL)	33.5	31.7-35.3
Reticulocyte (%)	1.1	0.5-2.5
Platelet count (10³/μL)	320	158-348
PT (%)	88	70-130
APTT (sec)	37.4	24-34
Fibrinogen (mg/dL)	580	200-400
FDP (μg/mL)	3.3	0-5
TSH (μIU/mL)	1.74	0.61-4.23
FT4 (ng/dL)	1.07	0.7-1.48
ALP (U/L)	77	38-113
AST (U/L)	20	13-30
ALT (U/L)	34	7-23
CPK (U/L)	35	41-153
LDH (U/L)	126	1124-222
γ-GTP (U/L)	41	9-32
T-CHO (mg/dL)	144	142-248
CHE (U/L)	189	201-421
Na (mEq/L)	141	138-145
K (mEq/L)	4.2	3.6-4.8
Cl (mEq/L)	103	101-108
Ca (mg/dL)	9.1	8.8-10.1
Creatinine (mg/dL)	0.58	0.46-0.79
BUN (mg/dL)	7.5	8-20
UA (mg/dL)	4.2	2.6-5.5
Glucose (mg/dL)	87	73-109
TP (g/dL)	7	6.6-8.1
Albumin (g/dL)	3.4	4.1-5.1
T-Bil (mg/dL)	0.42	0.4-1.5
Fe (μg/dL)	18	40-188
Hemoglobin A1c (%)	5.5	4.9-6
ESR (mm/hr)	77	3-15
IgG (mg/dL)	1248	861-1747
IgA (mg/dL)	193	93-393
IgM (mg/dL)	148	50-269
Ferritin (ng/mL)	128	12-60
CRP (mg/dL)	6.25	0-0.3
C3 (mg/dL)	153	73-138
C4 (mg/dL)	37	11-31
sIL2R (U/mL)	480	122-496
RF (IU/mL)	14	0-15
ASO (IU/mL)	157	0-250
ACE (U/mL)	7.6	7.0-25
Antinuclear antibody	×40	0-39
Toxoplasma IgM	(-)	0.00-0.99
Chlamydia trachomatis IgA	(-)	0-0.89
HIV	(-)	Negative
CMV-IgM (AU/mL)	(-)	0.00-0.84
CMV-IgG (AU/mL)	235.6	0.0-5.9
EBV VCA-IgM	(-)	0-9
EBV VCA-IgG	×320	0-9
EBNA	×40	0-9
T-SPOT.TB	(-)	Negative

**Figure 1:**
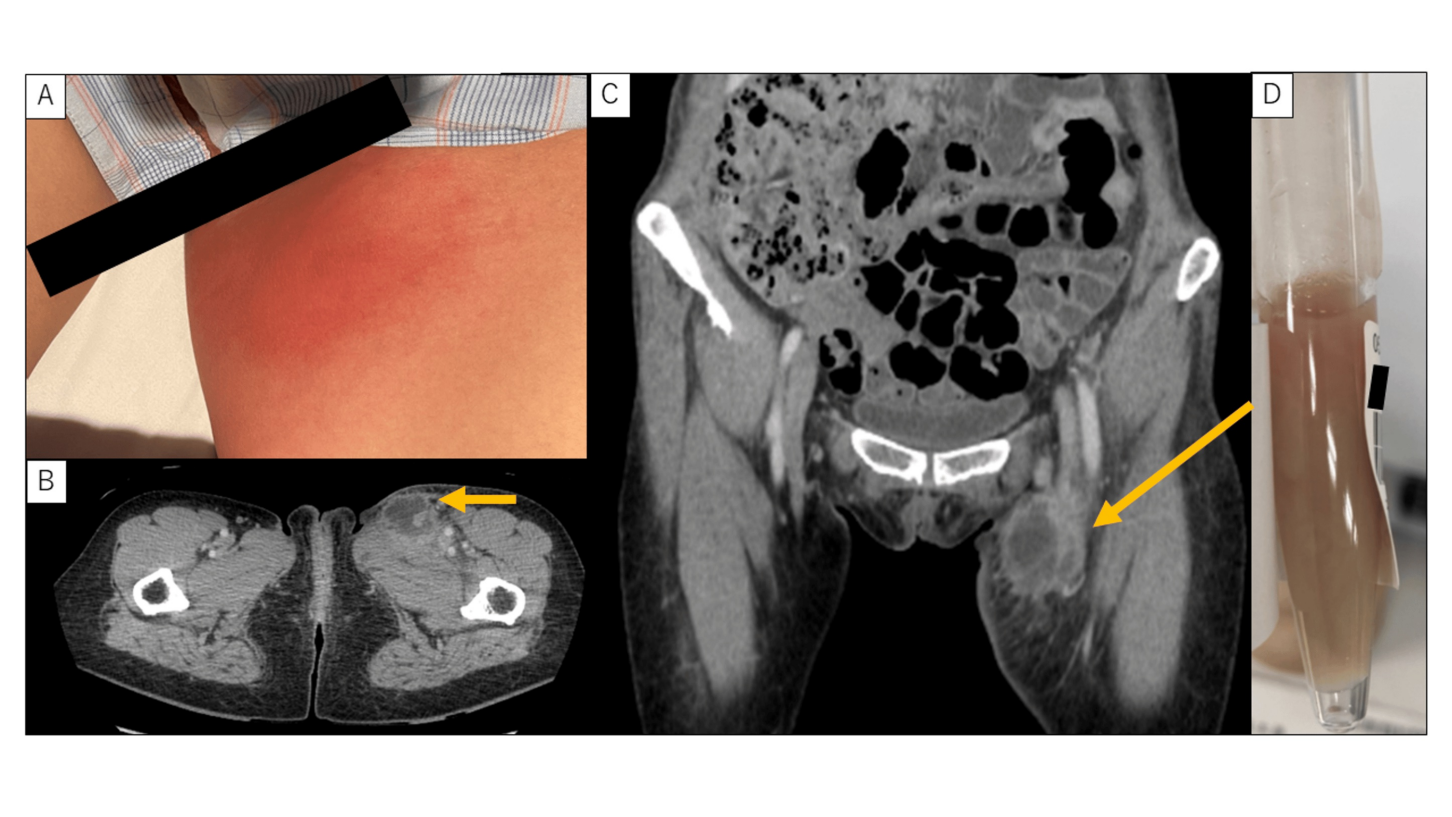
Clinical and radiologic findings of inguinal suppurative lymphadenitis (A) Gross appearance of the left inguinal region showing a fist-sized swelling. A firm, elastic lymph node was palpable with limited mobility. The overlying skin exhibited erythema and localized tenderness. (B) Contrast-enhanced sagittal computed tomography scan image of the pelvis. (C) Contrast-enhanced coronal computed tomography scan image showing an enlarged left inguinal lymph node with surrounding fat stranding. Within the node, a low-density area without contrast enhancement was observed, which is consistent with abscess formation. (D) Image of the purulent aspirate obtained via drainage of the left inguinal abscess. Approximately 8 mL of whitish, turbid pus was collected.

Contrast-enhanced computed tomography scan revealed significant enlargement of the left inguinal lymph node with surrounding fat stranding and a centrally hypoenhanced area, consistent with abscess formation (Figure 1A-1C). On day 15 of hospitalization (which is the day of diagnosis), ultrasound-guided needle aspiration yielded approximately 8 mL of thick, whitish purulent fluid (Figure 1D), which was sent for culture. Empiric treatment with intravenous ceftriaxone at a dose of 2 g/day was initiated. Due to minimal improvement on the following day, the ceftriaxone dose was increased to 4 g/day, resulting in symptomatic improvement within two days.

On day 21, culture of the abscess fluid yielded *S. pyogenes*. Meanwhile, the blood cultures had negative results. The antimicrobial susceptibility testing (Table 2) supported the use of step-down therapy to oral amoxicillin-clavulanate (250 mg/125 mg three times daily) combined with amoxicillin (250 mg three times daily).

**Table 2:** Antibiotic susceptibility profile of Streptococcus pyogenes isolated from inguinal abscess MIC: minimum inhibitory concentration; S: susceptible; R: resistant; PCG: penicillin G; ABPC: ampicillin; CTX: cefotaxime; CFPM: cefepime; CTRX: ceftriaxone; CFDN: cefdinir; FMOX: flomoxef; MEPM: meropenem; EM: erythromycin; CLDM: clindamycin; MINO: minocycline; CP: chloramphenicol; LVFX: levofloxacin; CPFX: ciprofloxacin; ST: sulfamethoxazole-trimethoprim; VCM: vancomycin

Antibiotic	MIC (μg/mL)	Interpretation
PCG	≤0.06	S
ABPC	≤0.12	S
CTX	≤0.12	S
CFPM	≤0.12	S
CTRX	≤0.12	S
CFDN	≤0.06	S
FMOX	0.12	S
MEPM	≤0.06	S
EM	>1	R
CLDM	>2	R
MINO	>4	R
CP	4	S
LVFX	>4	R
CPFX	>2	R
ST	0.5	S
VCM	0.5	S

**Figure 2:**
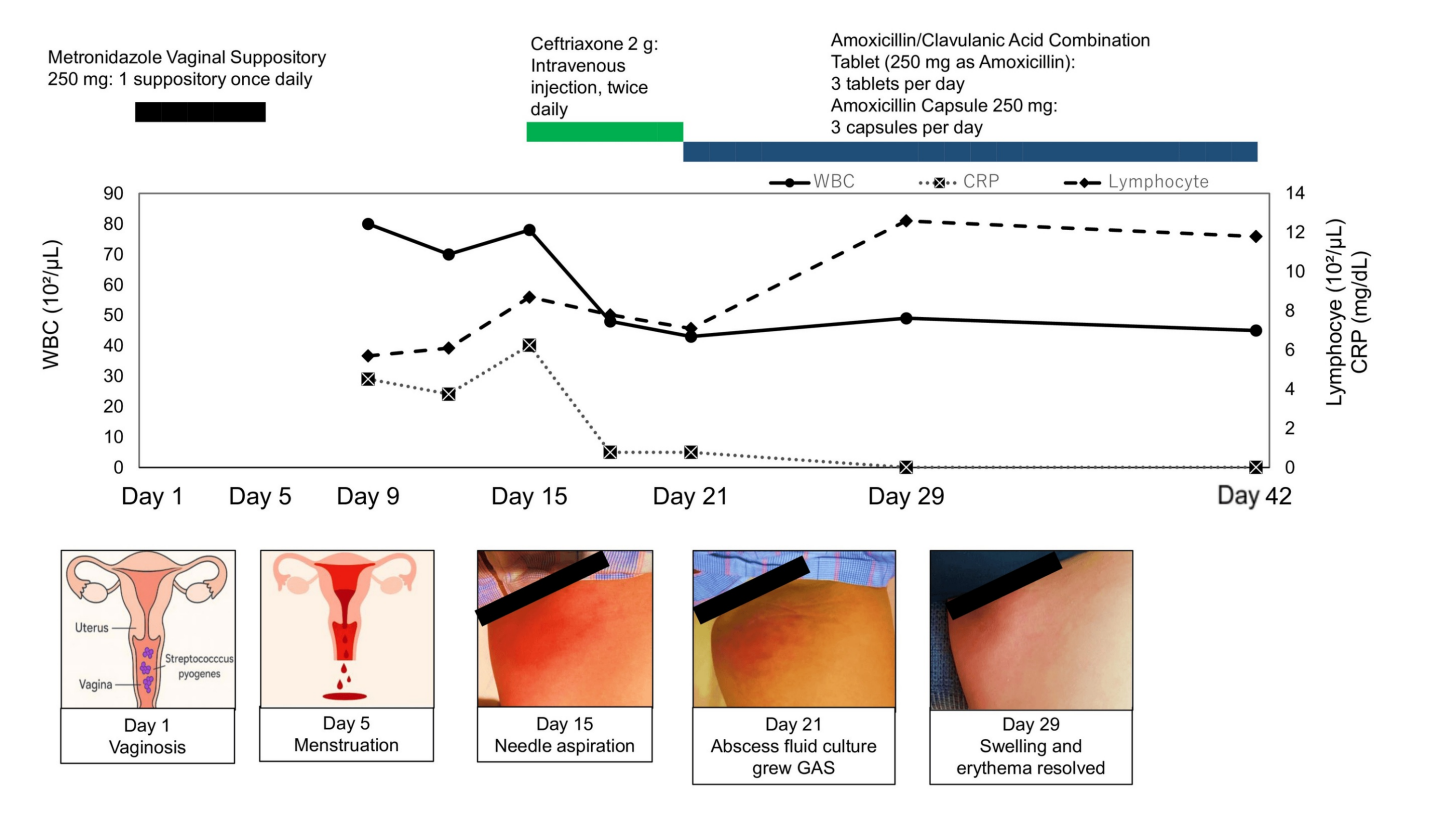
Timeline of the clinical course WBC: white blood cells; CRP: C-reactive protein; GAS: group A *Streptococcus* This figure is an original creation by the authors. The schematics under day 1 and day 5 were generated using an AI tool (ChatGPT-5.0, OpenAI, San Francisco, CA, USA) and further modified by the authors.

Swelling and erythema of the left inguinal region resolved by day 29 from vaginitis onset. Oral antibiotics were continued for four weeks, and the patient achieved complete recovery without sequelae by day 42, with resolution of lymphopenia (Figure 2).

## Discussion

GAS, which typically colonizes the pharynx or skin, is not a commensal organism of the vagina. Invasion of the female genital tract by GAS may occur due to the disruption of mucosal integrity caused by various factors such as menstrual cycle-related hormonal changes, alterations in vaginal pH, and depletion of *Lactobacillus*-dominant flora. Such changes can lead to the proliferation of *S. pyogenes* [2]. Recent studies show that the vaginal microbiome fluctuates across the menstrual cycle, with some women experiencing transient dysbiosis during menses, characterized by reduced *Lactobacillus* dominance and increased susceptibility to pathobiont overgrowth [5]. Such cycle-related changes may provide a window for GAS proliferation. In addition, metronidazole, commonly used for bacterial vaginosis, reduces anaerobes but does not effectively suppress streptococci or prevent recurrence, potentially leaving ecological niches for GAS persistence [6]. In this case, menstrual cycle-related changes and the use of intravaginal metronidazole might have altered the vaginal microbiota, leading to overgrowth and subsequent dissemination.

GAS possesses multiple virulence factors, such as M protein, C5a peptidase, and streptococcal oxidoreductases, that enhance tissue invasiveness and promote lymphatic spread (e.g., M protein causing phagocytosis evasion and bloodstream penetration) [7]. Superantigen-mediated immune activation and direct cytotoxic effects on lymphocytes have been implicated in the pathogenesis of invasive GAS, contributing to marked lymphopenia observed in several cases. Indeed, lymphopenia (<1,000/μL) has been reported in approximately 78% of invasive GAS infections [8]. In the current case, this hematologic change coincided with the period of rapid lymph node enlargement. The combination of lymphocyte depletion and virulence factor-driven immune dysregulation might have promoted both bacterial persistence and progression to suppurative lymphadenitis. Additionally, elevations in inflammatory biomarkers, including CRP, ferritin, and fibrinogen, were observed in this case, further reflecting the systemic inflammatory response associated with invasive GAS infection. To the best of our knowledge, there are no previous reports on purulent inguinal lymphadenitis caused by genital tract-associated GAS infection in a non-pregnant woman. Therefore, this case expands the recognized clinical spectrum of GAS disease and underscores the need for clinicians to consider this pathogen in acute inguinal lymphadenopathy, particularly if preceded by genital symptoms.

Inguinal lymphadenopathy refers to the enlargement of lymph nodes in the groin and can result from a wide range of etiologies. Common causes include reactive hyperplasia due to localized infections, such as lower limb cellulitis or sexually transmitted infections, as well as malignancies such as lymphoma or metastatic carcinoma. Clinically, acute inguinal lymphadenitis typically presents with tender, warm, and erythematous nodes, whereas lymphadenopathy due to malignancy often involves firm, matted, and nontender nodes. Therefore, inguinal lymphadenitis must be carefully distinguished from both infectious and malignant conditions. The most common pathogens include *Chlamydia trachomatis*, *Neisseria gonorrhoeae*, and *Treponema pallidum* [9]. In this patient, *Trichomonas vaginalis* and *C. trachomatis* were initially suspected, prompting empiric metronidazole therapy, which is not effective against GAS. Without timely pathogen identification, such infections may progress to abscess formation. In some cases, lymphadenitis resolves with antibiotics alone. However, cases complicated by abscesses often require drainage. Early aspiration under imaging guidance combined with targeted antibiotics can shorten recovery time and reduce the risk of recurrence [10-12]. In this case, prompt aspiration and appropriate antimicrobial therapy resulted in rapid clinical improvement and complete recovery.

## Conclusions

GAS-induced purulent lymphadenitis in adults is a relatively rare condition. However, it should be considered in the differential diagnosis of acute regional lymphadenopathy with fever. Imaging and aspiration for microbiological identification are important for diagnostic confirmation, and appropriate antibiotic therapy can achieve favorable outcomes. Nevertheless, further studies should be performed to validate the epidemiology, risk factors, and optimal management strategies of genital tract-associated GAS lymphadenitis.
